# Autistic traits as predictors of post-traumatic stress symptoms among patients with borderline personality disorder

**DOI:** 10.3389/fpsyt.2024.1443365

**Published:** 2024-09-02

**Authors:** Barbara Carpita, Chiara Bonelli, Vincenzo Schifanella, Benedetta Nardi, Giulia Amatori, Gabriele Massimetti, Ivan Mirko Cremone, Stefano Pini, Liliana Dell’Osso

**Affiliations:** Department of Clinical and Experimental Medicine, Section of Psychiatry, University of Pisa, Pisa, Italy

**Keywords:** autism spectrum disorder, autistic traits, borderline personality disorder, post- traumatic stress disorder, complex post-traumatic stress disorder

## Abstract

**Background:**

Autistic traits (AT) seem to be particularly frequent among patients with borderline personality disorder (BPD). Moreover, the autism spectrum is considered a vulnerability factor for the development of post-traumatic stress disorder (PTSD) symptoms, increasing the vulnerability of BPD subjects toward the development of a stress-related disorder.

**Aim:**

The study aimed to investigate the association between AT and trauma-related symptoms in a clinical sample of patients with BPD.

**Methods:**

A total of 48 patients with a clinical diagnosis of BPD and 52 healthy control (HC) subjects were recruited and assessed with the Adult Autism Subthreshold Spectrum Self-Report (AdAS Spectrum) questionnaire and the Trauma and Loss Spectrum—Self-Report questionnaire (TALS-SR). The BPD group was divided into two subgroups: BPD with a symptomatological diagnosis of PTSD (pBPD = 25) and BPD not diagnosed with PTSD (No-pBPD = 23).

**Results:**

The clinical sample scored significantly higher in almost all AdAS domains. Moreover, pBPD groups reported higher AdAS and TALS-SR scores in the total and in various domains than the No-pBPD group, which scored higher in several domains than HC. AdAS *Restricted interests and rumination* domain scores were positive predictors of BPD presence independently from PTSD, while *Inflexibility and adherence to routine* domain was a negative predictor. Finally, AdAS *Hyper/hyporeactivity to sensory stimuli* domain was a positive predictor only for inclusion in the pBPD group.

**Conclusion:**

Our study confirmed the existence of a statistically significant relationship between the autism spectrum and BPD, while BPD subjects diagnosed with PTSD seem to show a higher autism spectrum burden.

## 1 Introduction

Borderline personality disorder (BPD) is a pervasive pattern of instability in interpersonal bonds, affectivity, self-image, and strong impulsivity (1). The clinical picture is characterized by fear of being abandoned and intense efforts to avert the abandonment, unmotivated and intense anger with difficulty in being able to control it, self-injuries, paranoid ideations, and dissociative symptoms during periods of intense stress (1). According to recent literature, BPD represents 10% of outpatients, 15%–20% of inpatients, and 30%–60% of patients presenting with another personality disorder (2), with a prevalence in adult population ranging between 0.7% and 2.7% (3). According to biopsychosocial theories, BPD pathogenesis is linked to genetic and biological vulnerability, with deficits in emotional regulation, which lead to disorganized attachment patterns and early traumatic experiences (4), such as physical and emotional abuse (5, 6). In this perspective, traumatic experiences could play a central role in shaping the disorder explaining the frequent comorbidity with post-traumatic stress disorder (PTSD) (1). Moreover, BPD shows significant comorbidities with other psychiatric conditions such as bipolar, panic, and social anxiety disorder, feeding and eating disorders, and other personality disorders (1, 7–9).

Recently, a growing field of research is focusing on similarities between autism spectrum disorder (ASD) and BPD, revealing high rates of comorbidities between the two clinical conditions or even more between BPD and subthreshold autistic traits (AT) (10–15). This field of research was developed in the broader context of studies investigating female presentations of autism spectrum. While ASD is typically considered more prevalent among men, women with ASD without intellectual impairment were reported to show different kinds of specific interests and repetitive behaviors with respect to men as well as a generally milder impairment in social interactions, partially due to the use of social camouflaging strategies. This may lead to a more frequent under-recognition of ASD in women, who will receive instead a diagnosis of other mental disorders that are considered more likely to occur in women, ranging from anorexia nervosa to social anxiety and BPD (12, 13, 16, 17). According to a recent systematic review, the prevalence of BPD in ASD individuals ranges between 0% and 12%, while the overall prevalence of BPD in samples with ASD is 4% (18). Indeed BPD and ASD share common features such as impaired relationships and superficial friendships, emotional acts preferred to verbalization of emotions, deficits in verbal and nonverbal communication, impulsive and self-injurious behaviors, and alterations in social (12) functioning. Moreover, some studies stressed a similar deficit in recognizing, discriminating, and integrating emotions, ascribable to both ASD and BPD, as well as similar empathic deficits and impaired theory of mind (ToM), frequently related with aggressive and impulsive behaviors (12). Noticeably, both disorders have been linked to immune system and microbiota deregulation (19–21). On the other hand, the two disorders show some differences in diagnostic criteria such as the presence of limited and repetitive behaviors, activities, or interests and sensory hypo- or hyper-reactivity typical of ASD and not required in BPD. Conversely, the pervasive pattern of instability and the feeling of constant emptiness are not necessary criteria for ASD criteria (1). Furthermore, the emotional instability can be triggered by alterations in sameness or excessive sensory or cognitive stimulation in ASD disorder, while in BPD it is caused mainly by unstable and tumultuous relational boundaries (12).

As confirmed by several studies, the presence of subthreshold or full-blown ASD may lead to the structuring of a PTSD symptomatology. Indeed deficit in emotional and social reciprocity and empathy alterations may, on one hand, increase the risk of being exposed to traumatic experiences, especially relational ones. On the other hand, inflexibility and ruminative thinking may make the subject less able to successfully cope and adjust with the event or even to ask for help, enhancing the risk of developing trauma and stress-related symptoms also after life events of minor intensity with respect to those described by Criterion A of DSM-TR (10–15). Moreover, BPD patients often have a history of traumatic events occurring since childhood (4, 5). In this framework, the presence of underlying AT could be a central element for increasing the vulnerability of BPD subjects toward the development of a stress-related disorder, leading frequently to the structuring of a complex PTSD (cPTSD), whose symptomatology largely overlaps with BPD (13, 15, 22). cPTSD is induced by repeated exposure to minor traumas or traumatic events from which it is difficult to escape, leading to a clinical picture characterized by the typical PTSD symptom clusters associated to a deficit in self-organization (13). While the two conditions could be comorbid, cPTSD and BPD share several symptoms, such as negative self-image, persistent deficits in self-organization, feeling defeated and worthless, and deficits in interpersonal relationships (13). For this reason, several studies suggested the possibility that an unrecognized autism spectrum in childhood, especially in women, where this diagnosis is considered less common, eventually promoting the onset of PTSD or cPTSD symptoms after relational traumatic events, may lead to a diagnosis of BPD in adulthood (14).

In this framework, our study aimed to investigate the association between AT and trauma-related symptoms in a clinical sample of patients with BPD.

## 2 Methods

### 2.1 Study sample and procedures

The sample was composed of 100 participants divided into two groups: 52 healthy subjects (HC) belonging to medical and paramedical staff and 48 patients with a clinical diagnosis of BPD. The BPD patients were recruited at the psychiatric clinic of the “Azienda Ospedaliera Universitaria Pisana” among in- and out-patients who received a clinical diagnosis of BPD. The Structured Clinical Interview for DSM-5, Research Version (SCID-5-RV) (15), administered by trained clinicians, was used to ascertain the absence of mental disorders among HC. Subjects under 18 years old or over 65 years old, with poor compliance, persistent psychotic symptoms, unable to give informed consent, with mental disabilities, and language or intellectual disabilities that hindered examination were excluded from the recruitment. Every participant was given comprehensive information about the study and was given the chance to ask questions prior to completing a written informed consent form. The local ethical committee authorized all of the procedures, and the study was conducted in line with the Declaration of Helsinki.

### 2.2 Psychometric scales

All subjects were assessed with the AdAS Spectrum questionnaire and the Trama and Loss Spectrum—Self-Report (TALS-SR) questionnaire.

#### 2.2.1 The AdAS spectrum questionnaire

The AdAS Spectrum SR lifetime is a 160-question self-report scale, designed by Dell’Osso et al. in 2017, that explores autism spectrum symptoms in adults, including atypical symptoms, personality traits, and subthreshold patterns. Each item can be coded as “absent” (0) or “present” (1) (23). The questionnaire is divided into seven domains, namely:

1. Childhood and adolescence

2. Verbal communication

3. Non-verbal communication

4. Empathy

5. Inflexibility and adherence to routine

6. Restricted interests and ruminations

7. Hypo/hyper-reactivity to stimuli (23)

The questionnaire also features two cutoffs: for the presence of significant AT (43) and for the presence of clinical ASD symptoms (71) (24).

#### 2.2.2 The TALS-SR

The Trauma and Loss Spectrum (TALS-SR) was developed by Dell’Osso et al. in 2009, including 116 items that explore a range of loss and/or traumatic events and symptoms, behaviors, and personal characteristics that could represent manifestations and/or risk factors for the development of a stress response syndrome (25). It is divided into nine domains, namely:

1. Loss events domain

2. Reactions to bereavement domain

3. Potentially traumatic events

4. Reaction to losses

5. Re-experiencing

6. Avoidance and numbing

7. Maladaptive coping

8. Arousal

9. Personal characteristics (25)

As described in previous studies, the TALS-SR can be used to evaluate the fulfilment of DSM-5 symptomatological criteria for PTSD on the basis of an algorithm which includes the TALS-SR items evaluating those symptoms (15).

### 2.3 Statistical analyses

A chi-square analysis was used to investigate significant differences for categorical variables among the three groups. One-way analysis of variance (ANOVA) and Bonferroni test for *post-hoc* comparisons were performed to compare scores reported at the AdAS Spectrum and TALS-SR questionnaires among groups. Pearson’s correlation coefficient was used to evaluate the presence of significant correlations between AdAS and TALS-SR domains and total scores in the sample. Moreover, we conducted a multinomial logistic regression aiming to investigate which AdAS Spectrum domains may be predictive of the inclusion in the diagnostic groups, considering HC as the standard category. Finally, linear regression analyses were used to evaluate the possible predictive role of AdAS Spectrum total score (independent variable) on TALS-SR total score (dependent variable). The same analysis was conducted including AdAS Spectrum domain scores as independent variables. All analyses were performed with Statistical Package for the Social Sciences (SPSS) version 24.

## 3 Results

The sample was composed of 37 men and 63 women with a mean age of 33.81 ± 11.143 years.

Among patients in the BPD group, 25 reported a symptomatological diagnosis of PTSD according to the TALS-SR algorithm, while no caseness was found in the HC group, resulting in a significantly higher presence of PTSD in the BPD group (see **Table 1A**). On the basis of this data, we further divided the BPD sample in patients with and without a symptomatological diagnosis of PTSD according to TALS-SR. We named the first group pBPD (*n* = 25) and the second No-pBPD (*n* = 23).

**Table 1A:** Comparison of the presence/absence of a symptomatological diagnosis of PTSD among BPD and HCs.

Diagnosis		TSD presence	PTSD absence	Chi-square	*p*
BPD (*n* = 48)	*N*	25	23	36.111	<.001
	% in BPD	47.9	52.1		
	% in PTSD	100	30.7		
No BPD (HC) (*n* = 52)	count	0	52		
	% in BPD	0	100		
	% in PTSD	0	69.3		

No significant age and sex differences were reported in the three groups (see **Table 1B**).

**Table 1B:** Comparison of socio-demographic variables among groups.

		HC group (*n* = 52) Median ± SD	pBPD group (*n* = 25) Median ± SD	No-pBPD group (*n* = 23) Median ± SD		*p*
Age (years)		33.62 ± 12.265	32.76 ± 9.580	35.39 ± 10.290	*F* = .346	.708
Sex						
Male	Percentage	22	8	7	Chi-square = 1.322	.516
	% (diagnosis)	42.3	32	30.4		
	% (sex)	59.5	21.6	18.9		
Female	Percentage	30	17	16		
	% (diagnosis)	57.7	68	69.6		
	% (sex)	47.6	27	25.4		

The one-way analysis of variance (ANOVA) and Bonferroni test for *post-hoc* comparisons showed that pBPD subjects scored significantly higher than HC in all AdAS Spectrum domains. Significant differences were found between the two diagnostic groups, with a significantly higher score in patients with pBPD, with the exception of *Childhood/Adolescence*, *Restricted interests and rumination*, and *Inflexibility and adherence to routine*. In these domains, pBPD subjects scored higher but not significantly different than the subjects in the No-pBPD group, who also reported higher scores than HCs in almost all AdAS Spectrum domains with the exception of *Empathy* and *Inflexibility and adherence to routine* (see **Table 2**). Moreover, pBPD showed higher scores than No-pBPD and HC in all TALS-SR domains and total score, without a significant difference in *Grief reaction* between BPD and No-pBPD. In turn, No-pBPD patients showed significantly higher scores on all TALS-SR domains and total scores than HCs (see **Table 3**).

**Table 2:** Comparison of scores reported on AdAS Spectrum among groups.

AdAS Spectrum scores	HC group Mean ± SD	No-pBPD group Mean ± SD	pBPD group Mean ± SD	*F*	*p*
Child/adolesc.	3.50 ± 2.50	6.09 ± 3.10	8.04 ± 4.37	18.126	<.000^a^
Verbal comm.	2.36 ± 2.28	4.83 ± 2.81	6.64 ± 3.73	20.631	<.000^b^
Non-verbal comm.	4.94 ± 3.78	8.09 ± 4.83	11.32 ± 5.35	17.769	<.000^b^
Empathy	1.75 ± 1.81	2.78 ± 2.11	4,56 ± 3.06	13.225	<.000^c^
Inflex. and adherence to routine	7.96 ± 5.26	10.43 ± 5.06	14.52 ± 7.63	10.470	<.000^d^
Restricted interests and rumination	3.15 ± 3.16	7.00 ± 4.14	9.08 ± 5.09	21.137	<.000^a^
Hyper/hyporeact. to sensory inputs	1.31 ± 1.51	3.00 ± 2.92	5.44 ± 4.12	19.752	<.001^b^
Total score	24.98 ± 15.42	42.22 ± 18.54	59.60 ± 27.37	26.872	<.000^b^

*p* <.05.

a pBPD, No-pBPD > HC.

b pBPD > No-pBPD > HC.

c pBPD > No-pBPD, HC.

d pBPD > HC.

**Table 3:** Comparison of scores reported on TALS-SR questionnaire among groups.

TALS-SR Spectrum score	HC group Mean ± SD	BPD group Mean ± SD	pBPD group Mean ± SD	*F*	*P*
Loss events	3.27 ± 1.40	4.48 ± 1.50	5.79 ± 1.25	5,91	<.000^a^
Grief reactions	6.11 ± 4.40	13.17 ± 5.73	14.88 ± 5.36	19.61	<.000^b^
Pot. traum. events	2.19 ± 1.70	5.04 ± 3.21	7.09 ± 2.70	35.549	<.000^a^
Reaction to losses or upsetting events	3.83 ± 2.65	7.14 ± 3.64	11.08 ± 2.84	52.469	<.000^a^
Re-experiencing	1.13 ± 1.51	3.52 ± 2.27	5.80 ± 2.14	54.374	<.000^a^
Avoid. & numb.	1.15 ± 1.74	3.00 ± 2.15	7.64 ± 2.08	95.710	<.000^a^
Maladaptive coping	.13 ± .34	1.91 ± 1.83	4.32 ± 2.19	74.210	<.000^a^
Arousal	.42 ± .77	2.00 ± 1.54	3.52 ± 1.19	69.560	<.000^a^
Pers. charact./risk factors	.98 ± 1.26	3.13 ± 1.36	3.28 ± 1.46	34.871	<.000^a^
Total score	34.30 ± 18.74	40.74 ± 20.06	20.39 ± 14.46	24.71	<.000^a^

*p* <.05.

a pBPD > No-pBPD > HC.

b pBPD, No-pBPD > HC.

Pearson correlation analyses revealed that the AdAS Spectrum total and domain scores were significantly and positively correlated with TALS-SR domain and total in the total sample, with medium to strong correlations (see **Table 4**).

**Table 4:** Correlations between AdAS Spectrum and TALS-SR scores in the total sample.

AdAs domains/ TALS-SR domains	Child./adolesc.	Verbal comm.	Non-verbal comm.	Empat.	Inflex. & rout.	Rest. inter. & rum.	Hyper/hyporeac.	Total score
Loss events	.392*	.421*	.338*	.350*	.313*	.353*	.354*	.423*
Grief reac.	.496*	.453*	.502*	.376*	.503*	.545*	.497*	.586*
Pot. traum. ev.	.523*	.552*	.465*	.454*	.319*	.374*	.450*	.523*
Reac. losses	.478*	.515*	.548*	.414*	.423*	.520*	.444*	.573*
Re-exp.	.493*	.468*	.533*	.415*	.489*	.557*	.516*	.600*
Avoid. & numb.	.535*	.554*	.558*	.496*	.458*	.570*	.548*	.630*
Maladap. coping	.558*	.511*	.512*	.404*	.409*	.490*	.547*	.580*
Arousal	.492*	.468*	.493*	.435*	.459*	.557*	.541*	.589*
Pers. charac.	.507*	.456*	.508*	.304*	.490*	.573*	.539*	.590*
Total score	.578*	.593*	.579*	.455*	.522*	.595*	.496*	.670*

*p ≤.001.

A multinomial logistic regression with diagnostic groups as dependent variable (using HC group as the standard category) revealed the independent variable AdAS Spectrum total score as predictive for the inclusion in both pBPD and No-pBPD categories (see **Table 5**).

**Table 5:** Multinomial logistic regression analysis with group category as the dependent variable and AdAS Spectrum total score as the independent variable.

		B (SE)	Odds ratio	CI (95%)		*p*
				Upper limit	Lower limit	
BPD	Intercept	-2.588 (.599)	–	–	–	.000
	AdAS tot.	.054 (.016)	1.056	1.0891	1.024	.001
pBPD	Intercept	-.4.162 (.783)	–	–	–	.000
	AdAS tot.	.087 (.018)	1.091	1.129	1.054	.000

*R* ^2^ (Cox/Snell) = .346; *R* ^2^ (Nagelkerke) = .397.

Another multinomial logistic regression analysis was performed to evaluate which AdAS domains may be statistically predictive of the inclusion in one of the diagnostic categories considered as dependent variables with AdAS domains as independent variables. The results highlighted *Inflexibility and adherence to routine* and *Restricted interests and rumination* as significant predictors for inclusion in both the No-pBPD group and the pBPD group and *Hyper/hyporeact. to sensory inputs* as a positive predictor for pBPD inclusion (see **Table 6**). *Inflexibility and adherence to routine* was, in both groups, a negative predictor, while the other ones were positive predictors.

**Table 6:** Multinomial logistic regression analysis with group category as the dependent variable and AdAS Spectrum domains as independent variables.

		B (SE)	Odds ratio	CI (95%)		*p*
				Lower limit	Upper limit	
No-pBPD	Intercept	-2.164 (.692)	–	–	–	.002
	AdAS rout.	-.205 (.099)	.815	.672	.989	.038
	AdAs rum.	.377 (.146)	1.457	1.096	1.938	.010
pBPD	Intercept	-.3.535 (.853)	–	–	–	.000
	AdAS rout.	-.212 (.107)	.809	.656	.998	.048
	AdAS rum.	.365 (.156)	1.441	1.062	1.955	.019
	AdAS reatt.	.429 (.191)	1.535	1.056	2.231	.025

*R* ^2^ (Cox/Snell) = .346; *R* ^2^ (Nagelkerke) = .397.

Two linear regression analyses conducted with TALS-SR total score as dependent variable and AdAS Spectrum total (in one regression) and domains scores (in another regression) as independent variables reported that AdAS Spectrum total score and *Resticted interest interests and rumination* and *Hyper/hyporeactivity to sensory input* domains were statistically predictive of higher TALS-SR scores in the whole sample (see **Table 7**).

**Table 7:** Linear regression analysis with AdAS Spectrum domains and total scores as independent variables and TALS-SR total score as the dependent variable.

	B (SE)	BETA	*t*	*p*
Constant	14.269 (3.479)	-	4.102	.000
Child./adolesc.	1.036 (.674)	.173	1.538	.128
Verb. comm.	1.016 (.855)	.155	1.187	.238
Non-verb. comm.	.225 (.616)	.051	.365	.716
Empathy	.480 (.939)	.054	.511	.610
Inflex. & routine	-.442 (.482)	-.131	-.917	.362
Restrict. interest & rum.	1.495 (.655)	.308	2.283	**.025**
Hyper/hyporeact.	1.797 (.872)	.246	2.062	**.042**
*R* ^2^ = 0.708, adjusted *R* ^2^ = 0.501				
Total score constant	11.317 (3.068)	-	3.688	**.000**
Total score	.630 (.072)	.676	8.708	**.000**
*R* ^2^ = 0.676, adjusted *R* ^2^ = 0.457				

The bold values denote statistical significance at P < 0.05 level.

## 4 Discussion

This study aimed to investigate the relationship between AT and PTSD symptoms in a sample composed of 100 subjects divided into three groups: HC, BPD, and pBPD. According to our data, the two diagnostic groups reported significantly higher scores than HC in almost all AdAS Spectrum domains, confirming literature’s evidence about the link between autism spectrum and BPD: these results may support the hypothesis of the role of an underlying autism spectrum symptom as a vulnerability factor for developing BPD (10–16, 26). As expected, the BPD patients in our sample reported higher scores than HC in almost all TALS domains, with pBPD scoring significantly higher than No-pBPD except for *Grief and loss reaction.* While subjects with a full-fledged PTSD syndrome reported the highest scores, interestingly, the NO-pBPD group also reported higher TALS-SR scores than HC, confirming the presence of at least sub-threshold trauma and stress symptoms in all BPD patients (12). Moreover, the specific TALS-SR *Grief and loss reaction* domain explores reaction to loss: considering that the constant fear of being abandoned or rejected by significant others is a nuclear symptom of BPD, it is possible that this dimension could be present in BPD patients independently from the presence of a full-blown PTSD (1, 13).

Comparing the two diagnostic groups, pBPD subjects scored significantly higher than those belonging to the No-pBPD group except for *Childhood/adolescence* and *Restricted interests and rumination* domains where both groups scored higher than HC. These results seem to point out that elevated AT in childhood and tendency to rumination may be the autistic dimensions more linked to BPD independently from PTSD symptoms. According to our data, a recent meta-analysis reported diagnostic similarities between BPD and ASD such as relationship intensity and friendly superficiality, emotional acts preferred to verbalization of emotions, deficits in verbal and non-verbal communication, impulsive and self-injurious behaviors, alterations in social functioning, and rumination (13). On the other hand, the overall higher presence of AT among pBPD patients stresses the link between autism spectrum and vulnerability toward the development of PTSD, suggesting that BPD subjects with higher AT would be more likely to develop not only subthreshold stress-related symptoms but also a full-blown PTSD (14).

At the same time, *Restricted interests and rumination* domain was a significant predictor for inclusion in both diagnostic groups: this is in line with previous studies that stressed the role of ruminative thinking in the maladaptative cognition of BPD, while ruminative thinking could trigger and be triggered, in a vicious cycle, by negative feelings, eventually promoting behavioral dysregulation (1, 14, 27, 28). Noticeably, *Hypo/hyperreactivity to sensory stimuli* domain was a positive predictor only for pBPD inclusion. According to a growing field of literature, neuroatypical brains are characterized by hyper-perception, hyper-attention, hyper-memory, and hyper-emotionality, resulting in painful and excessive reactions to stimuli from the outside world, especially to traumatic events that are experienced with pain and constantly re-experienced (14, 29–31). Moreover, one recent study from our group, revealed that altered reactivity to stimuli was predictive of the development of post-traumatic symptoms in subjects with AT. In this perspective, the specific autistic dimension of altered reactivity to sensory input may increase the likelihood to develop a PTSD in BPD patients (14).

On the other hand, the *Inflexibility and adherence to routine* domain was a negative predictor for inclusion in both diagnostic groups. Indeed the traits investigated in this domain, such as routinariety, rigid habits, and inflexible cognition, may represent a compensatory factor with respect to the loss of control, impulsive behaviors, and emotions typical of BPD subjects. This result may be in line with a previous study stressing how inflexibility and adherence to routine may be a protective factor toward suicidality in BPD subjects (32).

Interestingly, our results reported a positive correlation between all AdAS domains and total score and TALS-SR in the total sample, confirming the close link between AT and post-traumatic dimension (13, 14). Particularly, *Resticted interests and rumination* and *Hyper/hyporeactivity to sensory input* domains were statistically predictive of higher TALS-SR scores. Ruminative thinking, for which the presence of AT could be a predisposing factor, may bring back to the mind traumatic experiences in a repetitive and intrusive way, blocking the processing of stressful triggers (33, 34). In this perspective, traumatic events, experienced with higher intensity, recur in a continuous re-experiencing that leads to the development of a post-traumatic stress symptomatology (13, 35–37).

This study should be considered in light of some limitations. First of all, the small sample size limits the extensibility of our results. Moreover the available sociodemographic variables were limited to sex and age. However, the homogeneity on sex and age in the groups limits the confounding factors related to these demographic featuress. Moreover, the high number of questionnaire items, requiring a long time to fill out the instruments, could increase the risk of inaccurate or hasty answers. Conversely, the large number of items allows us to explore a broad spectrum of symptoms, behaviors, and personality characteristics, providing an extremely detailed patients’ profile. Above all, AdAS and TALS-SR remain self-assessment that may lead patients to bias of symptoms’ overestimation/underestimation. It is also noteworthy to mention that, to evaluate clinically relevant PTSD symptoms, we used only one self-report questionnaire, the TAL-SR. Although the TALS-SR was used for this purpose in other studies (16), this methodology may have limited the accuracy of our findings. Finally, the cross-sectional design of the study, based on purely statistical predictivity, did not allow us to make an inference about cause–effect or temporal relationships. Further exploration through longitudinal research is needed to confirm our results.

Globally, our study confirmed the existence of a statistically significant relationship between AT and BPD as well as the role of AT in modulating the PTSD spectrum symptomatology in BPD patients. BPD patients with a symptomatic diagnosis of PTSD reported higher AT, suggesting a relationship between the severity of autistic symptoms and the risk of developing clinically relevant post-traumatic symptoms, which may be present at the subclinical level even in BPD subjects without a diagnosis of PTSD. Moreover, the ruminative dimension appears to be associated with BPD regardless of the presence of PTSD symptomatology, while autistic-like altered reactivity to sensory stimuli appears to be predictive of the development of PTSD in this population.

## Data Availability

The original contributions presented in the study are included in the article/supplementary material. Further inquiries can be directed to the corresponding author.
